# LncRNAs and regulated cell death in tumor cells

**DOI:** 10.3389/fonc.2023.1170336

**Published:** 2023-05-29

**Authors:** Yingying Wu, Xiaoling Wen, Yufang Xia, Xiao Yu, Yanhui Lou

**Affiliations:** Department of Gynecology, the Affiliated Hospital of Qingdao University, Qingdao, China

**Keywords:** lncRNAs, apoptosis, necroptosis, pyroptosis, NEtosis, entosis, ferroptosis, cuproptosis

## Abstract

Regulated Cell Death (RCD) is a mode of cell death that occurs through drug or genetic intervention. The regulation of RCDs is one of the significant reasons for the long survival time of tumor cells and poor prognosis of patients. Long non-coding RNAs (lncRNAs) which are involved in the regulation of tumor biological processes, including RCDs occurring on tumor cells, are closely related to tumor progression. In this review, we describe the mechanisms of eight different RCDs which contain apoptosis, necroptosis, pyroptosis, NETosis, entosis, ferroptosis, autosis and cuproptosis. Meanwhile, their respective roles in the tumor are aggregated. In addition, we outline the literature that is related to the regulatory relationships between lncRNAs and RCDs in tumor cells, which is expected to provide new ideas for tumor diagnosis and treatment.

## 1 Introduction

Cell death is a common physiological process that can be found in nearly all organisms. According to the Nomenclature Committee on Cell Death in 2018, cell death can be divided into Accidental Cell Death (ACD) and Regulated Cell Death (RCD) (1). ACD is an inevitable cell death due to physical breakdown of the plasma membrane caused by drastic external stimuli, such as chemical, physical, or mechanical stimuli, and this process cannot be biologically controlled (2). Differently, cell death regulated by drug or genetic intervention is named RCD that relies on precise signaling cascades and intracellular molecular mechanisms (3). RCDs can be divided into apoptosis, necroptosis, lysosome-dependent cell death (LDCD), pyroptosis, NETotic cell death (NETosis), immunogenic cell death (ICD), entotic cell death (entosis), PARP-1-dependent cell death (parthanatos), ferroptosis, autophagy-dependent cell death (ADCD/autosis), oxeiptosis, pH-dependent cell death (alkaliptosis), cuproptosis etc. (1). Usually, one signal transduction pathway can regulate a variety of RCDs, and a certain RCD can be regulated by multiple signal pathways. Therefore, blocking some important molecules on the signal transduction pathways can inhibit the occurrence of cell death (4).

Cancer is a major global public health problem. Due to cancer incidence and mortality increasing rapidly all over the world, cancer becomes one of the leading causes of death worldwide (5). Cancer has a high mortality rate and poor prognosis, which is closely related to the unlimited proliferation ability of tumor cells (6). The ability of tumor cells to proliferate indefinitely depends on the inhibition of cell death in the cells, such as imbalance of pro-apoptotic versus anti-apoptotic systems, and loss of p53 gene function (7, 8).

Long non-coding RNAs (lncRNAs) are RNAs of more than 200 nucleotides in length that lack open reading frames and do not encode proteins. In recent years, the role of lncRNAs in regulating biological functions and cellular activities has gradually been discovered. The mechanisms of lncRNAs have been reported which include interaction with DNA, RNA, and proteins (9–11). LncRNAs are involved in the development and progression of many diseases, especially the progression of tumors. LncRNAs play complex and precise regulatory roles in biological processes such as tumor cell proliferation, migration, invasion and drug resistance, etc. (12, 13). More than one study has reported that multiple RCDs occurring within tumor cells can be regulated mediated by lncRNAs (14, 15).

Recently, many scholars have conducted in-depth studies on the generation mechanisms of lncRNAs in regulating different RCDs in tumor cells and have made remarkable progress. These have certain guiding significance for the diagnosis and treatment of tumors. In this paper, we focus on the regulatory relationships between apoptosis, necroptosis, pyroptosis, NETosis, entosis, ferroptosis, autosis, cuproptosis and lncRNAs in tumor cells in recent years, hoping to provide new ideas for tumor diagnosis and treatment.

## 2 Apoptosis

### 2.1 Mechanisms of apoptosis

Apoptosis plays a significant role in tissue differentiation, organ development and aging, and clearance of injured or mutant cells. When cells undergo apoptosis, their morphology is mainly characterized by cell contraction, chromatin condensation, apoptotic body formation, and DNA fragmentation (1). The core mechanism of apoptosis is the activation of caspases, which are a large class of cysteine proteases that act in a cascade manner, of which caspase-3, -7, -8, and -9 play important roles in apoptosis (16). The two common activation pathways of apoptosis are the intrinsic pathway (also called mitochondrial pathway) and extrinsic pathway (also called death receptor pathway).

The initiation of the intrinsic pathway is caused by internal stimulus such as oxidative stress and genetic damage in cells. The intrinsic pathway is closely regulated by B cell lymphoma-2 (Bcl-2) family proteins, which are divided into two major classes: pro-apoptotic proteins (e.g., Bax, Bak, and Bid) and anti-apoptotic proteins (e.g., Bcl-2, Bcl-xL, and Bcl-w) (17). When the balance of Bcl-2 family proteins is broken, Bax and Bak form an opening in the outer mitochondrial membrane and induce changes in mitochondrial outer membrane permeability (MOMP) (18). Cytochrome c (Cytc) enters the cytoplasm through the mitochondrial intermembrane space and assists the pro-apoptotic factor, apoptotic protease activating factor 1 (APAF-1), in functioning to form apoptotic bodies (19). At the same time, when MOMP events occur in cells, caspase-9 binds to APAF-1 and is activated. And then caspase-9 further activates downstream caspase-3, ultimately triggering apoptosis (20).

The extrinsic pathway is activated by the combined action of different tumor necrosis factor (TNF) family members, including Fas receptor, tumor necrosis factor receptor (TNFR), TNF-related apoptosis-inducing ligand receptor (TRAILR), which are initiated by binding with their corresponding ligands. After binding to each other, these factors become type II membrane proteins with homotrimeric structures. They cleave the cell membrane to form soluble forms which can increase cell membrane permeability (21). The death-inducing signaling complex (DISC) is formed intracellularly, which includes the Fas-associated death domain (FADD), TNFR1-associated death domain (TRADD), and pro-caspase-8, after binding to the TNF family ligands. DISC can activate caspase-8 intracellularly and promote its maturation, which in turn activates downstream caspase-3 and caspase-7 and triggers apoptosis (22). In hepatocytes and fibroblasts, caspase-8 activates the pro-apoptotic protein Bid, and then induces Cytc release from mitochondria which leads to the initiation of the intrinsic pathway (23).

**Figure 1:**
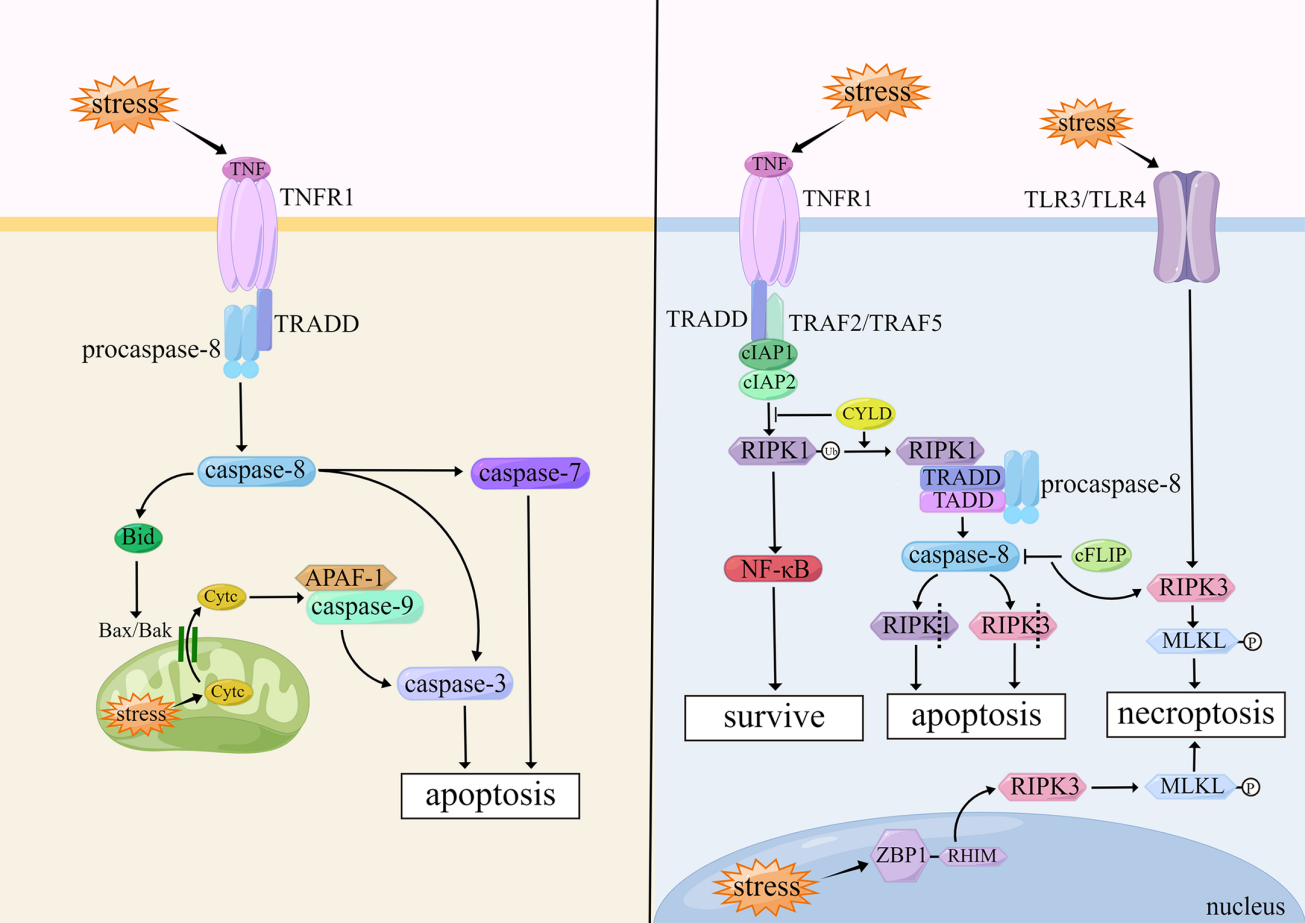
Mechanisms of apoptosis and necroptosis.

There is also a less common apoptosis within cells: the endoplasmic reticulum (ER) stress-induced apoptosis. When events occur in cells, such as hypoxia, oxidative damage, Ca^2+^ imbalance and viral infection, misfolded or unfolded proteins in the ER will increase, allowing the stress signal of the ER to be transmitted from the ER membrane to the nucleus, and promoting apoptosis (24). There are three apoptotic pathways induced by ER stress: IRE1/ASK1/JNK pathway, caspase-12 kinase pathway, and CHOP/GADD153 pathway. All three pathways play important roles in ER stress-induced apoptosis (25). (**Figure 1**)

### 2.2 Apoptosis in tumor cells

In tumor cells, the imbalance between the regulation of apoptosis and anti-apoptosis system often results in its antagonistic effects, promoting and suppressing cancer, and thus affecting cells survival. Anti-apoptosis, as an acquired feature of tumor cells, gives tumor cells survival advantages and promotes tumor evolution, growth and drug resistance (26). It has been reported that under apoptotic stress, cells release the growth factor FGF2, which rapidly up-regulates Bcl-2 and triggers the Bcl-2 dependent MEK-ERK pathway, thereby protecting neighboring cells from apoptosis (27). Similarly, apoptotic cells attract macrophages and polarize them into a regeneration-activated state to become tumor-associated macrophages (TAMs) in tumors. TAMs have the potential to promote tumor evolution and progression through multiple pathways. TAMs release anti-inflammatory mediators such as TGF-β1, IL-10 and PGE2, which promote tumor growth, activate angiogenesis, and promote tumor invasion and metastasis through proliferative signaling, regeneration and repair responses, and anti-tumor immune silencing of TAMs (28). For example, in aggressive non-Hodgkin lymphoma, apoptotic cells highly express genes encoding growth factors and angiogenesis, which can provide nutrients to the tumor microenvironment and promote TAMs accumulation, tumor growth and angiogenesis (29). However, how the balance between apoptosis and anti-apoptosis in tumor cells is disrupted, and how more elaborate mechanisms are regulated has yet to be elucidated, which require us to conduct further studies.

### 2.3 Apoptosis and lncRNAs in tumors

LncRNAs can induce or inhibit apoptosis of tumor cells by regulating key molecules of apoptosis. One study has suggested that lncRNA MNX1-AS1 up-regulates the expression of Bcl-2 by sponging miR-6785-5p in gastric cancer cells, which can promote proliferation, migration and invasion, and then inhibits apoptosis (14). LncRNA PCAT1 interacts with dyskerin pseudouridine synthase-1 (DKC1) to activate the vascular endothelial growth factor (VEGF)/protein kinase B (AKT)/Bcl-2/caspase-9 pathway and promote the transcription and translation of Bcl-2, contributing to enhancing abilities in proliferation and invasion of non-small cell lung cancer cells and inhibiting apoptosis of tumor cells (30). Subsequent experiments revealed that lncRNA H19, LINC00662, lncRNA SNHG6, and LINC01087 can act on different miRNAs in cholangiocarcinoma, colon cancer, gastric cancer and glioma, respectively. However, these all lncRNAs cause increased expression level of Bcl-2, inhibition of apoptosis, and tumor progression ultimately (31–34). Among them, lncRNA H19 plays a role in sepsis, myocardial injury, and vascular smooth muscle injury in addition to acting in cholangiocarcinoma (35–37). LncRNAs can induce or inhibit apoptosis by regulating apoptosis-related molecules in the caspase family. Experiments performed by Pan et al. indicated that lncRNA PDPK2P can interact with 1,3-phosphoinositide‐dependent protein kinase-1 (PDK1) and regulate the development of hepatocellular carcinoma through PDK1/AKT/caspase-3 signaling pathway (38). PDK1 plays a crucial part in regulating the phosphorylation of AKT, which is involved in apoptosis thereby changing the states of cells (39). LncRNA PDPK2P promotes AKT phosphorylation and inhibits the expression of caspase-3 by interacting with PDK1, and then inhibits the apoptotic pathway of hepatocellular carcinoma cells and promotes proliferation of tumor cells (38).

LncRNAs are also involved in the regulation of the extrinsic pathway of apoptosis. It has been reported that lncRNA MAGI2-AS3 can inhibit breast cancer cell growth by increasing the expression of Fas and Fas ligand and promoting apoptosis (40). LncRNA MAGI2-AS3 inhibits tumor cell proliferation in hepatocellular carcinoma cells as well (41). LncRNA MAGI2-AS3 binds to miR-374b-5p and inhibits its expression, thereby playing a positive regulation part on genitalia family member-1 (SMG1), and increasing the expression levels of SMG1, which in turn plays a tumor suppressor role. SMG1 is a potential tumor suppressor that can antagonize mammalian target of rapamycin (mTOR) to regulate leukemia cell growth in acute myeloid leukemia (42). However, conclusions from studies regarding the effect of SMG1 on apoptosis are not entirely consistent. In response to Smac mimetic compounds (SMC), an experimental small molecule, SMG1 inhibited SMC-mediated TNF-α-induced apoptosis to protect cells. Downregulation of SMG1 expression promotes SMC-mediated TNF-α-induced caspase-8 activation (43).

Wang et al. revealed that when osteosarcoma cells undergo ER stress, LINC00629 expression is increased by activating the KLF4/LAMA4 pathway, and ER stress-induced apoptosis is inhibited to promote tumorigenesis and metastasis (44). Taken together, lncRNAs play a significant role in the regulation of various pathways of apoptosis. (**Table 1**)

**Table 1:** Regulation of RCDs by different lncRNAs in tumor cells.

LncRNA	Pathway	Function	RCDs	Disease	Reference
LncRNA MNX1-AS1	LncRNA MNX1-AS1/miR-6785-5p/Bcl-2 Axis	Inhibition	Apoptosis	Gastric Cancer	(14)
LncRNA PCAT1	LncRNA PCAT1/DKC1/VEGF/AKT/Bcl-2/caspase-9 Pathway	Inhibition	Apoptosis	Non-small Cell Lung Cancer	(30)
LncRNA H19	LncRNA H19/miR-612/Bcl-2 Axis	Inhibition	Apoptosis	Cholangiocarcinoma	(31)
LINC00662	LINC00662/miR-340-5p/ERK Signaling Pathway	Inhibition	Apoptosis	Colon Cancer	(32)
LncRNA SNHG6	LncRNA SNHG6/ miR-1297/Bcl-2 Axis	Inhibition	Apoptosis	Gastric Cancer	(33)
LINC01087	LINC01087/miR-384/Bcl-2 Axis	Inhibition	Apoptosis	Glioma	(34)
LncRNA PDPK2P	LncRNA PDPK2P/ PDK1/AKT/caspase-3 Signaling Pathway	Inhibition	Apoptosis	Hepatocellular Carcinoma	(38)
LncRNA MAGI2-AS3	LncRNA MAGI2-AS3/Fas/FasL Signaling Pathway	Promotion	Apoptosis	Breast Cancer	(40)
LncRNA MAGI2-AS3	LncRNA MAGI2-AS3/miR-374b-5p/Smg1 Signaling Pathway	Promotion	Apoptosis	Hepatocellular Carcinoma	(41)
LncRNA MAGI2-AS3	LncRNA MAGI2-AS3/miR-374b-5p/SMG1/mTOR Pathway	Promotion	Apoptosis	Acute Myeloid Leukemia	(42)
LINC00629	LINC00629/KLF4/LAMA4 Pathway	Inhibition	Apoptosis	Osteosarcoma	(44)
LncRNA H19	LncRNA H19/miR-675/p-MLKL/RIP3 Axis	Promotion	Necroptosis	Liver Cancer	(56)
LncRNA PVT1	LncRNA PVT1/ZBP1/EZH2 Axis	Promotion	Necroptosis	Hepatocellular Carcinoma	(57)
LncRNA NEAT1	LncRNA NEAT1/miR-296-5p/GSDMD Axis	Inhibition	Pyroptosis	Glioma	(76)
LncRNA NEAT1	LncRNA NEAT1/miR-448/GSDME Axis	Inhibition	Pyroptosis	Colorectal Cancer	(77)
LncRNA XIST	LncRNA XIST/miR-335/NLRP3 Axis	Promotion	Pyroptosis	Non-small Cell Lung Cancer	(79)
LncRNA ADAMTS9-AS2	LncRNA ADAMTS9-AS2/miR-223-3p/NLRP3 Axis	Promotion	Pyroptosis	Gastric Cancer	(80)
LncRNA AP	LncRNA AP/NADPH/GSH/ROS Axis	Promotion	NETosis	Colorectal Cancer	(106)
LncRNA OR3A4	LncRNA OR3A4/NADPH/ROS Axis	Inhibition	NETosis	Osteosarcoma	(107)
LncRNA KTN1-AS1	LncRNA KTN1-AS1/KTN1/Rho GTPase Axis	Inhibition	Entosis	Bladder Cancer	(122)
LncRNA NORAD	LncRNA NORAD/miR-125a-3p/RhoA Axis	Inhibition	Entosis	Pancreatic Cancer	(124)
LncRNA NORAD	LncRNA NORAD/CXCR4/RhoA/ROCK Signaling Pathways	Inhibition	Entosis	Lung Cancer	(125)
LncRNA NORAD	LncRNA NORAD/Rho GTPase Pathway	Inhibition	Entosis	BreastCancer	(126)
LncRNA PVT1	LncRNA PVT1/miR-214-3p/GPX4 Axis	Inhibition	Ferroptosis	Liver Cancer	(141)
LncRNA GABPB1-AS1	LncRNA GABPB1-AS1/GABPB1/ROS Axis	Promotion	Ferroptosis	Hepatocellular Carcinoma	(143)
LncRNA NEAT1	LncRNA NEAT1/SLCA11/ROS Axis	Promotion	Ferroptosis	Non-small Cell Lung Cancer	(135)
LncRNA H19	LncRNA H19/miR-19b-3p/FTH1 Axis	Promotion	Ferroptosis	Lung Cancer	(15)
LncRNA H19	LncRNA H19/mTOR Signaling Pathway	Inhibition	Autosis	Glioma	(162)
LncRNA H19	LncRNA H19/mTORC1/4E-BP1 Axis	Promotion	Autosis	Pituitary Cancer	(163)
LncRNA CASC9	LncRNA CASC9/AKT/mTOR Pathway	Inhibition	Autosis	Oral Squamous Cell Carcinoma	(164)
LncRNA HAGLROS	LncRNA HAGLROS/miR-100-5p/mTOR Signaling Pathway	Inhibition	Autosis	Gastric Cancer	(165)
LncRNA EPIC1	LncRNA EPIC1/Myc/AKT-mTORC1 Signaling Pathway	Inhibition	Autosis	Breast Cancer and Ovarian Cancer	(166)
LncRNA MIR31HG	LncRNA MIR31HG/miR-193a-3p/TNFRSF21 Axis	Inhibition	Cuproptosis	Lung Adenocarcinoma	(184)
LncRNA XIST	LncRNA XIST/miR-92b-3p/MTF1 Axis	Promotion	Cuproptosis	Breast Cancer	(188)

## 3 Necroptosis

### 3.1 Mechanisms of necroptosis

Necroptosis belongs to a nonapoptotic cell death independent of the caspase pathway. Necroptosis differs from necrosis. Necroptosis can be induced by stimulation with superfamily members of TNFR, pattern recognition receptor (PRR), T cell receptor, and chemotherapeutic agents; but necrosis is considered an “accidental” death that is not regulated by molecular events (45). Necroptosis is morphologically similar to necrosis, which is characterized by loss of integrity of the plasma membrane, swelling of the cytoplasm and organelles, chromosome condensation, and release of cellular contents (46).

Necroptosis is associated with activation of receptor-interacting protein-1 (RIP1), receptor-interacting protein-3 (RIP3), and mixed lineage kinase domain-like protein (MLKL), of which RIP3 and MLKL are considered biomarkers of necroptosis (47). Upon binding of TNF and TNFR1 on cell membranes, the complex I is formed, including receptor-interacting protein kinase 1 (RIPK1), TRADD, cellular inhibitor of apoptosis-1/2 (cIAP1/2), TNFR-related factor 2/5 (TRAF2/5), which in turn stimulates different signaling pathways and induces cell death (48). When RIPK1 is polyubiquitinated by cIAP1/2, the nuclear factor-κB (NF-κB) pathway is activated and cells survival predominate. However, when RIPK1 is deubiquitinated by cylindromatosis (CYLD), the NF-κB pathway is suppressed, forming the complex II consisting of RIPK1, TRADD, caspase-8, and FADD. Caspase-8 inactivates RIPK1 and RIPK3 by proteolytic cleavage, which leads to apoptosis. When caspase-8 is inhibited by cell FLICE-like inhibitory protein (cFLIP), RIPK3 phosphorylates its substrate, MLKL, to form p-MLKL. P-MLKL oligomerizes and translocates to the plasma membrane, ultimately leading to necroptosis (49). Toll-like receptor 3/4 (TLR3/TLR4) on the cell membrane can also initiate RIPK3-mediated necroptosis after lipopolysaccharide (LPS) stimulation (50).

Furthermore, there is an inside-out death pathway in cells. Z-DNA-binding protein-1 (ZBP1/DAI/DLM-1) in the nucleus is a nucleic acid sensor containing a RIP homotypic interaction motif (RHIM). Upon viral stimulation, RHIM can recruit and activate RIPK3, thereby initiating RIPK3- and MLKL-dependent necroptosis, which contributes to nuclear envelope breakdown, DNA leakage into the cytoplasm, and cell death eventually (51). (**Figure 1**)

### 3.2 Necroptosis in tumor cells

In tumors, foci of cell death (i.e., tumor necrosis) are often present due to conditions such as hypoxia and nutritional deficiencies (46). Necroptosis plays a very complex part in tumor cells. For one thing, down-regulating expression of necrosis factors allow tumor cells to resist necroptosis. Necroptosis-induced inflammatory reactions can also promote tumorigenesis and metastasis. For another, necroptosis can trigger strong immune responses in tumor cells, induce cell death, and then protect against tumor progression (45). MLKL, RIPK1, and RIPK3 have all been revealed to be down-regulated in breast cancer, rendering tumors unable to recruit immune cells. Consequently, immune surveillance is evaded and cancer-promoting effects are exerted (52). Accumulation of large amounts of RIPK3 in intestinal epithelial cells brings about severe necroptosis and promoting colon carcinogenesis (53). When tumor cells are glucose deficient, ZBP1 and its downstream pathways will be activated. RIPK3 expression increases, and necroptosis of tumor cells is induced, but cell metastasis is promoted as well (54). Additionally, in ovarian cancer, RIP1 has a dual role, both promoting cancer cell proliferation and reducing tumor resistance of drug thereby promoting the anticancer effects of chemotherapeutic agents such as cisplatin (55). However, in tumor cells, it still is an open question how the carcinogenic and anticancer effects of these necrosis factors are coordinated, and the mechanism behind it needs further study.

### 3.3 Necroptosis and lncRNAs in tumors

LncRNAs can regulate necroptosis through different pathways in tumor cells and improve or reduce tumor proliferation, invasion and migration. LncRNA H19 is the upstream of miR-675, and the expression levels of the two are positively correlated. MiR-675 can downregulate FADD and inhibit apoptosis; however, miR-675 has the ability to induce significant upregulation in p-MLKL and RIP3, which triggers necroptosis (56). A study collectively suggested that upregulation of lncRNA PVT1 is associated with elevated expression of necroptosis-related proteins, such as ZBP1, RIPK3, and p-MLKL (57). Enhancer of Zeste Homolog-2 (EZH2) negatively correlates with lncRNA PVT1 in hepatocellular carcinoma cells, and lncRNA PVT1 can increase methylation of the ZBP1 promoter and promote necroptosis by combining with DNA methyltransferase-1 through EZH2 (57, 58). However, in studies on breast cancer, promoter hypermethylation is found to lead to downregulation of ZBP1 expression, which promoted growth and migration of tumor cells (59).

Necroptosis-associated lncRNAs perform increasingly vital regulatory roles in many different tumors. In a key study by Luo et al., LINC00460, LINC02773, CHROMR, LINC01094, FLNB-AS1, ITFG1-AS1, LASTR, and LINC01638 are analyzed in TCGA database, which are highly expressed in gastric adenocarcinoma cells, while REPIN1-AS1, UBL7-AS1, PINK1-AS, and PVT1 are lowly expressed. These lncRNAs together regulate necroptosis in gastric adenocarcinoma cells (60). LncRNAs such as AP003392.3, AL928654.1, AL133371.2, AC007991.4, AC011445.1, and LINC00996 have been associated with necroptosis in ovarian cancer studies (61).

## 4 Pyroptosis

### 4.1 Mechanisms of pyroptosis

Pyroptosis is an inflammatory cell death mediated by the gasdermin superfamily that is triggered by the activation of certain inflammasomes and presents a form of DNA damage distinct from apoptosis (62). When pyroptosis occurs, the cytomorphology shows the following changes: the cell membrane forms transmembrane pores under inflammatory stimulation, and the cell permeability increases, resulting in cell swelling and osmotic lysis, intact nuclei but fragmented chromatin, and the release of intracellular pro-inflammatory components (63).

The gasdermin superfamily consists of gasdermin A/B/C/D/E (GSDMA/B/C/D/E) and DFNB59, of which GSDMD is of great importance to induce pyroptosis (64). Most inflammasomes contain three parts: nucleotide-binding domain, leucine-rich-repeat-containing receptors (NLRs), pro-caspase-1 and apoptosis-associated speck-like protein containing a caspase activation and recruitment domain (ASC) which consists of pyrin domain (PYD) and caspase activation and recruitment domain (CARD). The NLRP3 inflammasome, containing PYD, as an important cytosolic PRR, is activated upon TLR recognition of stimuli such as infection. NLRP3 recruits ASC via PYD-PYD domain interaction, and then activates pro-caspase-1 via CARD-CARD domain interaction (65). Activated caspase-1 can stimulate the activation of pro-IL-1β, pro-IL-18 and GSDMD cleavage N-terminal domain of GSDMD (N-GSDMD) appears oligomerization, inducing the formation of holes on the cell membrane, promoting cell membrane rupture, while releasing inflammatory factors such as IL-1β and IL-18, which leads to the occurrence of pyroptosis ultimately (66). It has been documented that caspase-8 can be activated by inflammasomes in caspase-1-deficient cells (67). Pyroptosis mediated by caspase-8 is independent of GSDMD, which belongs to a delayed, alternative form of cell death involving inflammasomes, accompanied by the release of massive mature IL-1 which has a protective function in the host (67).

**Figure 2:**
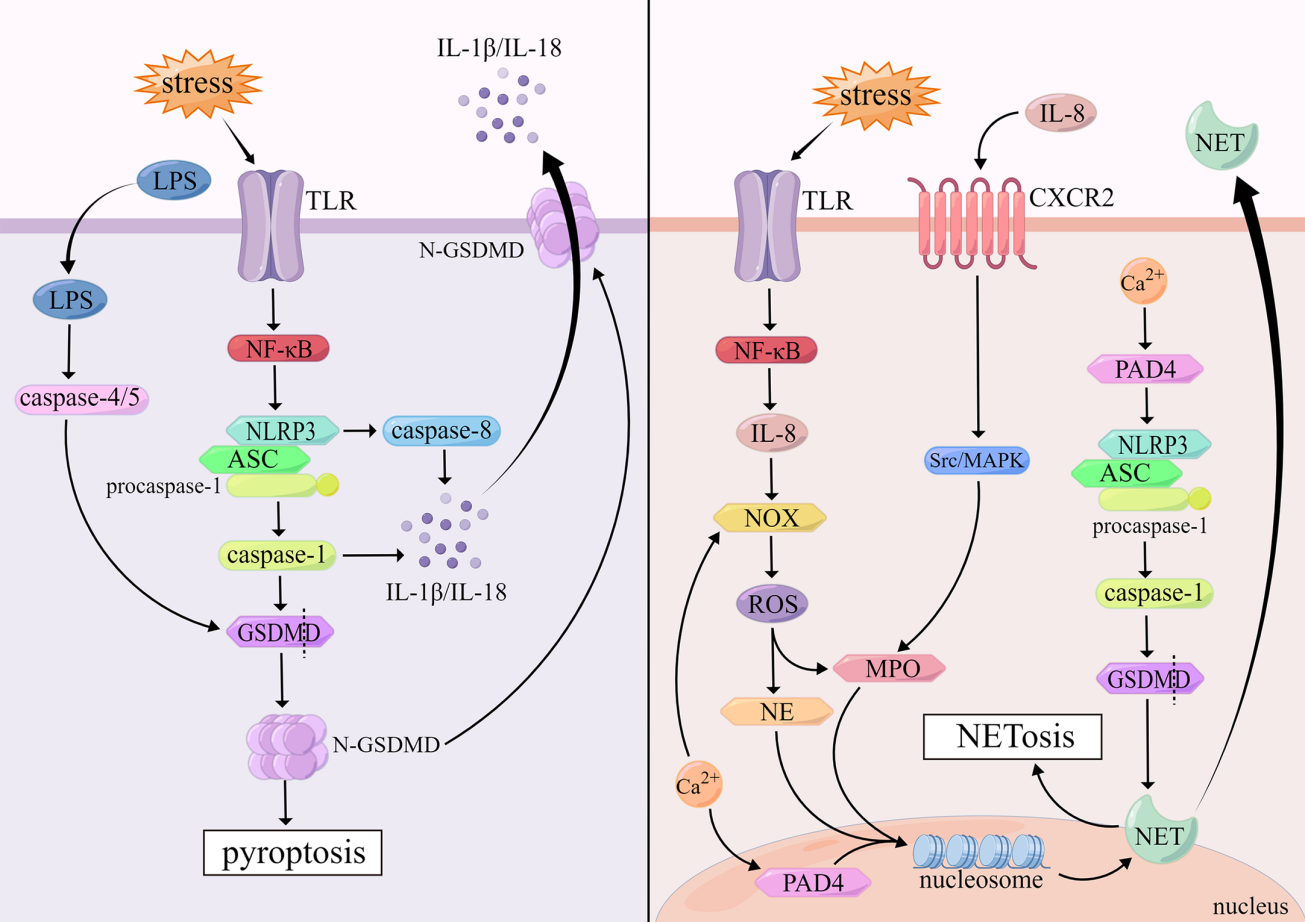
Mechanisms of pyroptosis and NETosis.

There is also a pyroptosis pathway induced by caspase-4/5 in human cells. Caspase-4/5 can be activated by direct binding to the CARD at the N-terminus of LPS, which in turn cuts GSDMD into N-GSDMD. And then, N-GSDMD is oligomerized and transferred to the cell membrane to induce the assembly of NLRP3 Inflammasomes, and pyroptosis occurs finally (68). (**Figure 2**)

### 4.2 Pyroptosis in tumor cells

Pyroptosis is a double-edged sword in tumors. On the one hand, it can inhibit the occurrence and development of tumors; on the other hand, it can form a microenvironment suitable for the growth of tumors (69). For example, as an critical link in the development of pyroptosis, high expression of GSDMD in some tumors can improve the survival time of patients, while high expression of GSDMD often leads to poor prognosis in adrenocortical carcinoma, chromophobe renal cell carcinoma and other tumors (70). GSDMD expression is decreased in gastric cancer tissues, and S/G_2_ cell transition is accelerated by activating signal transducer and activator of transcription-3 (STAT3) and phosphatidylinositol 3-kinase (PI3K)/AKT signaling pathway to regulate cell cycle-related proteins and promote tumor proliferation in vitro and in vivo (71). Besides GSDMD, GSDME also plays different roles in tumor development. GSDME can enhance the phagocytosis of TAMs and the number and function of tumor lymphocytes, activating caspase-independent pyroptosis in target cells to inhibit tumors and enhance anti-tumor immunity (72). However, some studies run counter to the conclusions above. GSDME-mediated pyroptosis can promote the release of high-mobility group box-1 (HMGB1) to induce the proliferation of tumor cells and the development of colorectal cancer (73). Although the research on pyroptosis is getting deeper and deeper, the outcome brought about by pyroptosis on tumors still needs us to continue to explore.

### 4.3 Pyroptosis and lncRNAs in tumors

Recent advances have begun to clarify that molecules in the mechanism of pyroptosis (e.g., GSDM) are regulated by lncRNAs. Knockdown of lncRNA PVT1 significantly decreased the production and release of inflammatory factors, while the expression of GSDMD and caspase-1 is inhibited, which gives rise to inhibition of pyroptosis (74). LncRNA MALAT1 inhibits miR-141-3p, which in turn promotes the expression of GSDMD and induces pyroptosis (75). One study has demonstrated that lncRNA KCNQ1OT1, LINC01278, lncRNA MIRLET7BHG and lncRNA NEAT1 can act as upstream targets of miR-296-5p and regulate the occurrence and development of glioma (76). Among them, lncRNA NEAT1 can inhibit pyroptosis by regulating GSDMD (76). However, findings by Su et al. showed that down-regulated lncRNA NEAT1 suppresses pyroptosis through sponging miR-448 to regulate GSDME expression levels in ionizing radiation-induced colorectal cancer cells (77).

LncRNAs also regulate pyroptosis through other molecules in the pyroptosis mechanisms. For instance, lncRNA KCNQ1OT1 reduces pyroptosis by targeting miR-214-3p and caspase-1 (78). In non-small cell lung cancer, lncRNA XIST increases the expression of mitochondrial superoxide dismutase-2 through sponging miR-335; while knockdown of lncRNA XIST promotes reactive oxygen species (ROS) production and NLRP3 inflammasomes activation, resulting in triggering pyroptosis (79). Overexpressed lncRNA ADAMTS9-AS2 can downregulate miR-223-3p and activate NLRP3 inflammasomes in gastric cancer, which promotes the development of pyroptosis (80).

Wang et al. analyzed the database that lncRNA ZFPM2-AS1, lncRNA KDM4A-AS1, lncRNA LUCAT1, lncRNA NRAV, AL031985.3, AL049840.5, lncRNA MKLN1-AS, AC099850.3 and LINC01224 promote pyroptosis in hepatocellular carcinoma, while lncRNA CRYZL2P-SEC16B and AC008549.1 play a protective role and inhibit pyroptosis (81). AC006001.2, LINC02585, AL136162.1, AC005041.3, AL023583.1, and LINC02881 are associated with the development of ovarian cancer and involved in regulating pyroptosis in ovarian cancer (82). LncRNAs involved in the regulation of pyroptosis have been reported in different tumors such as breast cancer, prostate cancer, glioblastoma, head and neck squamous cell carcinoma, soft tissue sarcoma and so on, which are not repeated here (83–87).

## 5 NETosis

### 5.1 Mechanisms of NETosis

NETosis is a form of cell death that depends on neutrophil extracellular traps (NET) produced after neutrophil activation. NET is composed of DNA-histone complexes and cytotoxic proteins. NETs can not only capture invading pathogens, but also degrade them with NETs associated proteolytic enzymes (88, 89). Morphological changes in NETosis are characterized by chromatin decondensation accompanied by separation of the inner and outer layers of the nuclear membrane, fusion of the nucleoplasm and cytoplasm, cell size reduction, cell membrane rupture, NETs release, and ultimately cell death (90).

The mechanisms of neutrophil extracellular trapping net withering are divided into two types according to whether nicotinamide adenine dinucleotide phosphate (NADPH) is dependent or not. Cells attacked by viruses, bacteria and other pathogens activate TLR on the cell membrane, which initiates NF-κB signaling pathway, promotes the release of IL-8, and then induces NADPH-oxidase (NOX) activation and ROS production (91). ROS facilitates the release of antimicrobial proteins such as myeloperoxidase (MPO) and neutrophil elastase (NE) from neutrophils (92). These proteases induce chromatin decondensation in the nucleus, which is enhanced by MPO (93). Furthermore, IL-8 also activates downstream Src/MAPK signaling pathway through combining with CXC chemokine receptor-2 (CXCR2), which will promote the release of MPO (94). These proteins enter the nucleus, disrupt chromatin structure. The decondensed chromatin binds to proteins released by neutrophils to form NETs, and finally the cell membrane is destroyed, the cell ruptures, and intracellular NETs are released into the plasma (92). However, it remains unresolved how NETs are released from the nucleus into the cytoplasm through the nuclear envelope. One theory is that GSDMD mediates the nuclear envelope permeabilization, resulting in the release of NETs into the cytoplasm (95). Another perspective suggests that DNA is released from the nucleus into the cytoplasm via vesicles (96). In addition, neutrophil stimulation triggers the release of calcium stored in the ER. Upon intracellular calcium overload, NOX is also activated to stimulate the production of ROS, triggering NETosis that is not associated with infection (97).

NADPH-independent NETosis is mainly driven by peptidyl arginine deiminase-4 (PAD4) via citrullinated histones. Activation of PAD4 requires high concentrations of calcium (98). PAD4 is mainly localized in the nucleus of resting neutrophils, where it mediates citrullination of nucleosomal histone H3, resulting in reducing histone positive charge and the affinity between histones and negatively charged DNA, which brings about dissociation of histones from DNA and loss of chromatin structure, and induces NETs formation (96). In addition, PAD4 is involved in NLRP3-mediated ASC oligomerization and speckle formation in neutrophils, and then activates caspase-1 and its downstream molecule, GSDMD, touching off NETosis (99, 100). (**Figure 2**)

### 5.2 NETosis in tumor cells

NETs have been reported to exert carcinogenic and anticancer effects. Important components in NETs, such as MPO and protease, can inhibit tumor growth and metastasis and promote tumor cell death; however, some proteases in NETs can also degrade the extracellular matrix and promote tumor cell extravasation and metastasis (88). NETs also have the ability to awaken dormant tumor cells (101). There was a study showing that CXCR1 and CXCR2 agonists acting as major mediators of NETosis, interact with chemokines, CXC motif chemokine ligand 1/6/7/8 (CXCL1/6/7/8), induce NETs formation in tumors, and inhibit immune-mediated cytotoxicity (102). Studies using a nude mice model have suggested that CXCL1/2 knockdown significantly reduces tumor metastasis (103). NETs can directly alter the metabolic program of tumor cells to promote tumor growth as well, which is mainly due to NE released from NETs activating TLR4, increasing mitochondrial biogenesis related gene expression, increasing mitochondrial density, increasing ATP production, and accelerating tumor growth (104). A study has shown that the NETs content in the blood of patients with early stage of head and neck cancer is significantly higher than that of healthy people, while in the advanced stage of cancer, the NETs level is reduced (105). Therefore, NETs are of essential as cancer therapeutic targets to delve into the regulation of NETosis and the balance between NETs formation and destruction in tumor cells.

### 5.3 NETosis and lncRNAs in tumors

In recent years, there have not been many reports on the regulation of NETosis by lncRNAs, but studies have shown that lncRNAs are involved in regulating related molecules of NETosis. Pep-AP encoded by lncRNA AP can inhibit pentose phosphate pathway, reduce NADPH/NADP+ and glutathione (GSH) levels, promote ROS accumulation, induce redox imbalance, and thus inhibit colorectal cancer cell growth (106). In osteosarcoma, knockdown of lncRNA OR3A4 suppresses NADPH production and increases intracellular ROS, leading to ER stress and cell death (107). He et al. predicted five lncRNAs (AC079336.5, LINC00623, AC087752.4, AL645933.2, and LINC00426) to be associated with NETosis by database analysis, which affects cancer prognosis in head and neck squamous cell carcinoma (108).

## 6 Entosis

### 6.1 Mechanisms of entosis

Entosis was first proposed in 2007 by Overholtzer and colleagues (109). This cell death pattern occurs in tumor cells and epithelial cells when cells are embedded in vacuoles of host cells and degraded by lysosomal enzymes of host cells. Under the microscope, internalized cells are surrounded by host cells which contain large vesicles, forming cell-in-cell (CIC) structures, but the cell membrane integrity of both cells is not disrupted. Host cells are stretched and form narrow cytoplasmic areas with crescent-shaped nuclei (110). Garanina et al. described five stages of entosis through electron microscopy. In the first stage, the internalized cells are round and the nuclei remain round. In the second stage, the internalized cells contract and produce short protrusions extending from the cell body to the entotic vacuole membrane. During the third stage, internalized cells further decreases with irregular nucleus shape and accumulation of cytoplasmic vacuoles. At the fourth stage, the shape of the internalized cells and nuclei is further deformed. The accumulation of cytoplasmic vacuoles increases and the nucleoli disappear. In the fifth stage, the entotic cells contain only residuals (111).

**Figure 3:**
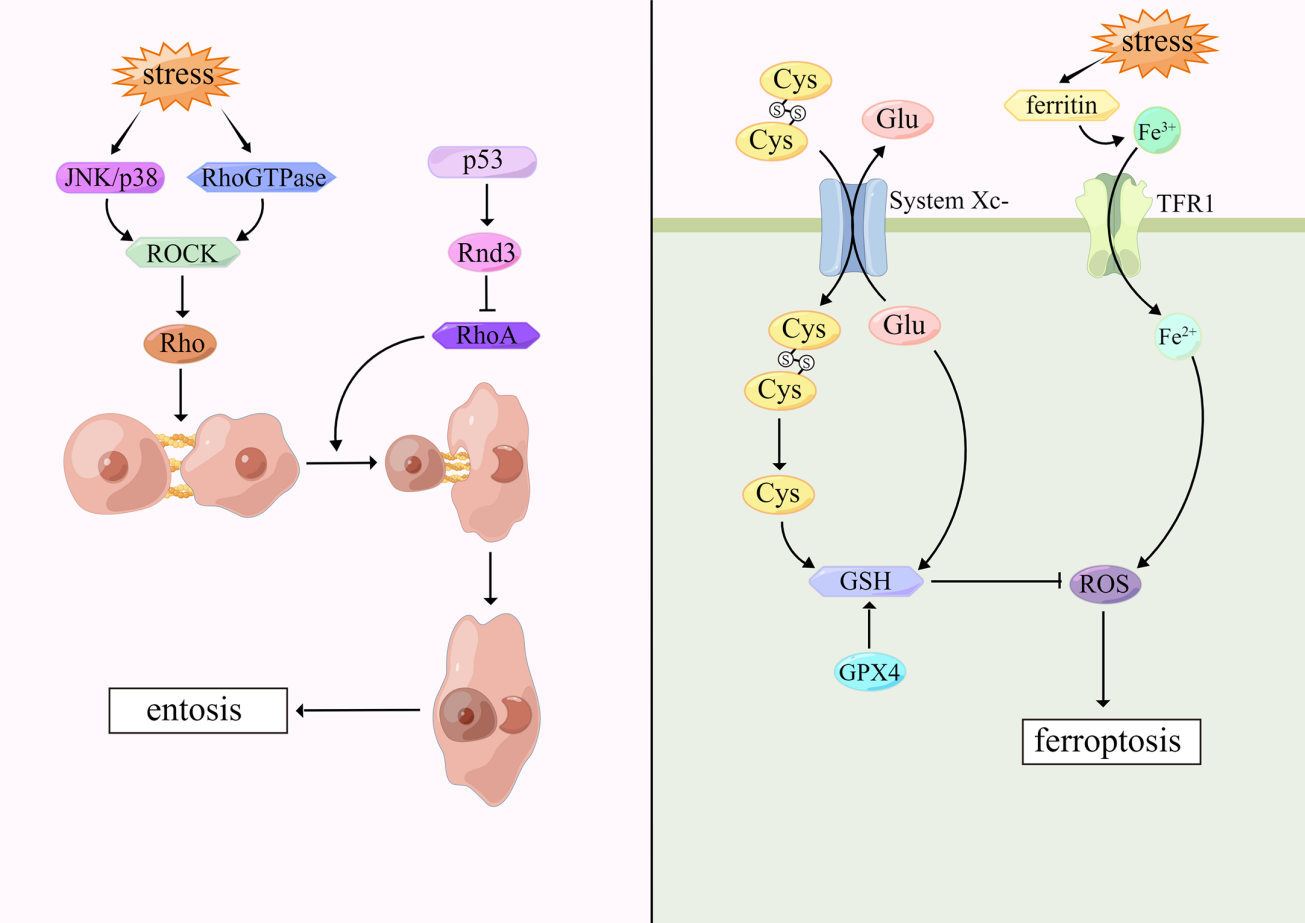
Mechanisms of entosis and ferroptosis.

Cell adhesion and cytoskeletal rearrangement play a central part in entosis (4). Recent evidence suggests that cell-cell junctions are involved by both E-cadherin and α-catenin and regulated by Rho guanosine triphosphatases (Rho GTPases) (112). Aneuploid mitotic arrest occurs when cells are exposed to external stimuli that cause DNA damage (113). Cells detach from the extracellular matrix. Rho-GTP activates the downstream effector molecule Rho-associated coiled-coil containing protein kinase (ROCK), and then induces the activation of Rho protein, which promote actin-myosin interaction and increase contractility. Due to the action of cadherin, cells are tightly connected to their adjacent cells, so that cells are internalized by adjacent cells, and encapsulated by lysosomes of host cells. Finally, internalized cells degrade and die through the lysosomal pathway mediated by LC3-associated phagocytosis (114). However, it has been suggested that P53 locally inhibits RhoA signaling and myosin contraction at cell-cell junctions by targeting Rnd3, which leads to asymmetric RhoA activation and thus promotes entotic CIC formation (113). UV radiation is also one of the triggers of entosis. Upon cells stimulated, JNK/p38 signaling pathway is activated, which triggers ROCK-dependent entosis (115). Most interestingly, the present study has demonstrated that entosis occurs when breast tumor cells are deficient in glucose in which AMPK plays an important role (116). (**Figure 3**)

### 6.2 Entosis in tumor cells

In many cancers, tumor malignancy and poor prognosis are the link with entosis which even acts as an escape mechanism to evade adverse factors from other cells, contributing to treatment failure or cancer recurrence (117). In high-grade clear cell renal carcinomas, CIC structures tend to indicate high malignancy and metastasis (118). While Durgan, J et al. found that epithelial cells can phagocytose and kill abnormally dividing cells to inhibit tumor growth when entosis happens (119). For example, TRAIL-induced entosis in colorectal tumor cells results in the appearance of CIC structures and poor prognosis (120). Entosis can exert anticancer effects in novel methylselenoesters-induced pancreatic cancer cells (121). Entosis plays a dual role of cancer, but there is no complete and systematic study on the antagonistic mechanism of these two effects, which suggests new ideas for future studies on entosis.

### 6.3 Entosis and lncRNAs in tumors

Rho GTPases are important links in entosis. LncRNAs have been reported to modulate Rho GTPases-mediated signaling and affect the survival of cells (122, 123). In bladder cancer, lncRNA KTN1-AS1 promotes tumor development by regulating the KTN1/Rho GTPase axis (122). An elegant study out of Li et al. examined that lncRNA NORAD competitively sponges miR-125a-3p, bringing about dysregulation of RhoA and migration and invasion of pancreatic cancer cells (124). LncRNA NORAD has also been shown to promote lung cancer cell proliferation, invasion, and migration through CXCR4 and RhoA/ROCK signaling pathway (125). According to latest bioinformatics research, lncRNA NORAD can also promote breast cancer progression through the Rho GTPase pathway (126). However, the research about lncRNAs and entosis in other tumors is still elusive.

## 7 Ferroptosis

### 7.1 Mechanisms of ferroptosis

Ferroptosis was first proposed by Dixon in 2012 (127). Morphologically, ferroptosis is characterized by mitochondria shrunk, mitochondrial cristae reduced or disappeared, mitochondrial membrane density increased and mitochondrial outer membrane ruptured, while nuclear structure is intact, without nuclear fissures and chromatin marginalization, as opposed to cellular morphological changes that occur in apoptosis and necroptosis (128). Biochemically, ferroptosis is characterized by iron-dependent lipid peroxidation and increased intracellular ROS (129).

Ferroptosis is based on iron accumulation and lipid peroxidation. Typically, excess iron is present as ferritin. However, when cells are stimulated internally or externally, ferritin is degraded and Fe^3+^ is released in large amounts, which is reduced to Fe^2+^ by transferrin receptor-1 (TFR1) and released into the iron pool in the cytoplasm, increasing ROS and causing destructive effects (130, 131). On the cell membrane, SLC7A11 and SLC3A2 are critical subunits of glutamate-cystine reverse transporter (System Xc-) which can mediate glutamate transport out of the cells, cystine transport into the cells. Intracellular cystine is reduced to cysteine that synthesizes reduced GSH (132). Glutathione peroxidase 4 (GPX4) is a selenocysteine enzyme which can regulate the sensitivity of cells to ferroptosis. GPX4, which is activated by cystine via mTOR complex 1 (mTORC1) signaling pathway, can detoxify lipid peroxidation in the presence of GSH, thus reducing lipid ROS to inhibit ferroptosis (133, 134). When the antioxidant system, especially the System Xc-/GSH/GPX4-dependent antioxidant defense system, is inactivated, lipid ROS accumulates and ferroptosis occurs. Erastin is a commonly used ferroptosis-inducing factor, which suppresses SLC7A11 expression rendering System Xc-dysfunction, and then inhibits cystine uptake, reduces GSH production, and produces ferroptosis (135). (**Figure 3**)

### 7.2 Ferroptosis in tumor cells

Since the concept of “ferroptosis” has been proposed, there have been increasing studies on the role of ferroptosis in tumor suppression. Ferroptosis can be activated or resisted through different signaling pathways to regulate tumor growth and drug resistance, such as hippo signaling pathway, PI3K/AKT/mTOR signaling pathway and so on (136). PARP inhibitors have been approved for use in ovarian and breast cancers. It has been suggested that PARP inhibitors promote ferroptosis by suppressing SLC7A11 in a p53-dependent manner. In combination with erastin, the sensitivity of PARP inhibitors can be improved in the treatment of ovarian cancer, thereby exerting anticancer effects (137). Like PARP inhibitors, STAT3 inhibitors down-regulate SLC7A11 and GPX4 to trigger ferroptosis, inhibit gastric cancer progression and reduce chemoresistance (138). Ferroptosis, in addition to anticancer effects, can also produce inflammation-related immunosuppression in the tumor microenvironment, which facilitates tumor growth. HMGB1 can bind to receptor for advanced glycation end-products (RAGE or AGER) to promote inflammatory responses in macrophages, and inhibition of HMGB1 may have potential therapeutic effects on ferroptosis-related diseases (139). Furthermore, AGER in pancreatic cancer mediates the uptake of oncogenic protein KRAS by macrophages, which ultimately leads to macrophage polarization and stimulates tumor growth (140). Ferroptosis plays an anticancerous role in most tumors and is still a major hotspot in the study of cancer treatment.

### 7.3 Ferroptosis and lncRNAs in tumors

With the growing knowledge of ferroptosis, the regulation between lncRNAs and ferroptosis is continuously being investigated and developed. It has been shown that high level of lncRNA PVT1 directly interacts with miR-214-3p, hindering adsorption of GPX4, increasing GPX4 content and maintaining GSH in a reduced state, which inhibits ferroptosis and promotes tumor cell proliferation (141). Under the induction of erastin, miR-214 increases with GSH decreasing, lipid oxidation enhancing and ROS production increasing, causing ferroptosis in hepatocellular carcinoma (142). Erastin can also up-regulate lncRNA GABPB1-AS1 which downregulates the protein level of GABPB1 by blocking the translation of GABPB1 mRNA. Due to a series of biological effects mediated by GABPB1, intracellular ROS is increased which promotes ferroptosis and prolongs the survival time of liver cancer patients (143). There has been a study reporting that silencing of lncRNA NEAT1 can act in combination with erastin to down-regulate SLCA11 and GPX4, induce ROS increase, and promote the upregulation of Bax and caspase-3, inhibit the expression of Bcl-2, which motivates ferroptosis and apoptosis, and inhibits tumor proliferation, metastasis and invasion in non-small cell lung cancer (135). In addition to lncRNA NEAT1, lncRNA H19 can also trigger ferroptosis in lung cancer via the miR-19b-3p/FTH1 axis (15).

LncRNAs can promote or suppress ferroptosis by regulating the expression of miRNAs or mRNAs which affect key molecules of ferroptosis. There are still many studies on ferroptosis mediated by lncRNAs, which have been reported in different tumors, such as ovarian cancer, prostate cancer, bladder cancer, acute myeloid leukemia and so on (144–147). However, in benign tumors, the role of lncRNAs in regulating ferroptosis remains to be explored.

## 8 Autosis

### 8.1 Mechanisms of autosis

According to published guidelines, we can classify cell death into three types by the tightness of the relationship between autophagy and cell death: autophagy-associated cell death, autophagy-mediated cell death, and autophagy-dependent cell death (148). In 2013, Beth Levine named autophagy-dependent cell death “autosis” (149). We focus here on autophagy-dependent cell death. When autosis occurs, the morphological features of cells are characterized by enhanced adhesion between cells and extracellular matrix, disruption or disappearance of ER structure, swelling around the nucleus, and mild chromatin condensation (149).

**Figure 4:**
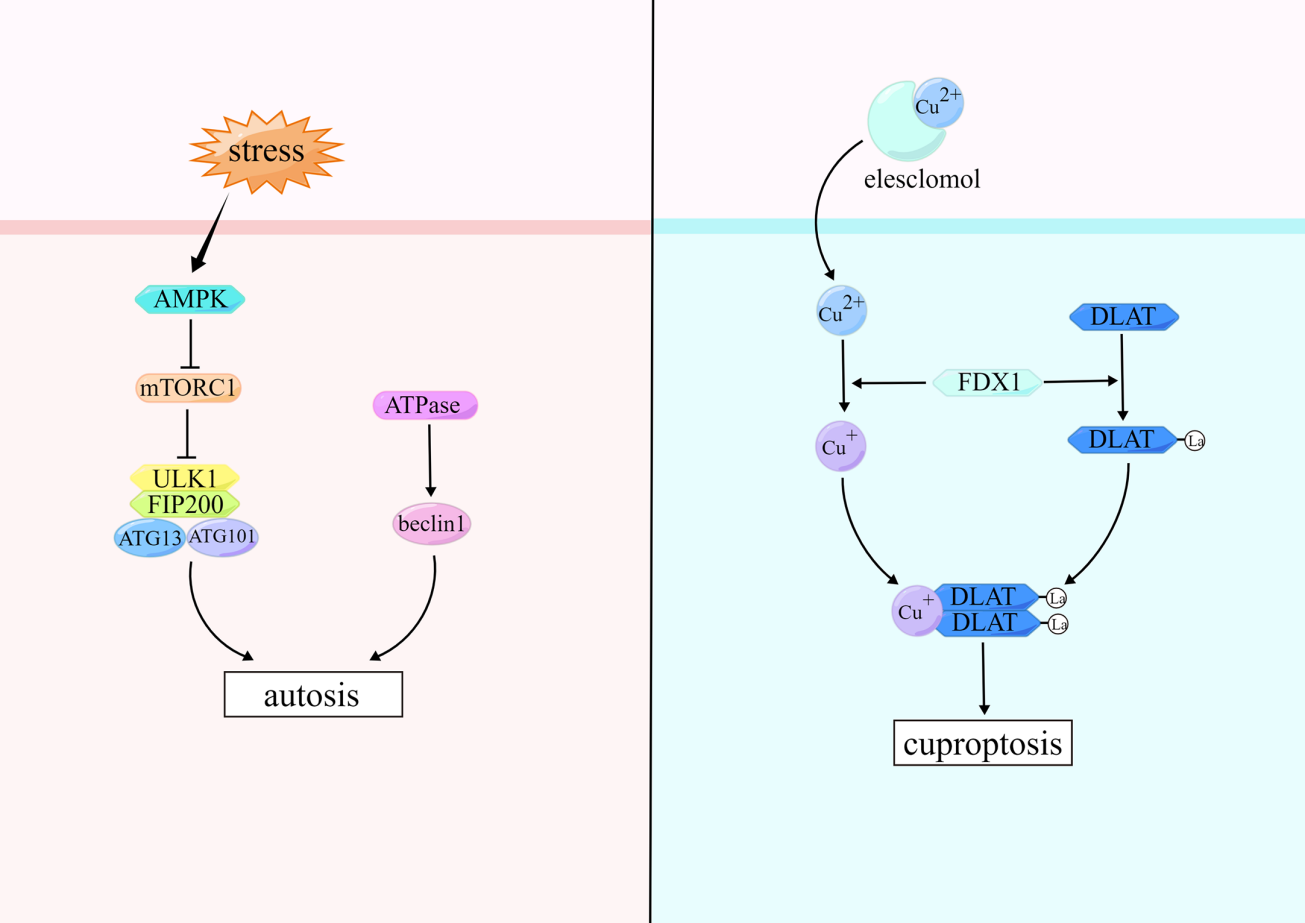
Mechanisms of autosis and cuproptosis.

Autosis usually occurs as a result of stimulation by high doses of autophagy-inducing peptides, starvation, and permanent cerebral ischemia, and the key to initiation is the Unc-51-like autophagy activating kinase 1 (ULK1) complex (150). ULK1 complex which is the core complex in autosis consists of ULK1, ATG13, ATG101, and FAK family kinase-interacting protein of 200 kDa (FIP200) (151). The ULK1 kinase complex recruits the PI3K complex to phagocytes, which catalyzes the conversion of phosphatidylinositol to phosphatidylinositol 3-phosphate (PI3P) and promotes autophagosomes formation. The PI3K complex consists of PI3K, the autophagy protein beclin-1 (BECN1), vacuolar protein sorting-15, and other partners (150). PI3K can interact with a variety of regulatory proteins to form different complexes that will selectively participate in different stages of autosis and initiate downstream cascade enzymatic reactions (152). MTORC1 regulates the ULK1 complex to inhibit autosis (153). The upstream molecule of mTORC1 is AMP-activated protein kinase (AMPK), which inhibits mTORC1 and induces autosis (154, 155). Moreover, it has been shown that Na+, K+-adenosine triphosphatase (ATPase) can promote autosis by activating BECN1 (156). (**Figure 4**)

### 8.2 Autosis in tumor cells

It is well-known that autophagy exists as a pro-survival stress response in most cases. Moreover, the above three types of autophagy are not clearly divided in most studies, and most of the studies are on autophagy-mediated cell death. However, autosis still plays an important role in inducing tumor cell death, especially as a spare cell death program in apoptosis-deficient tumors (157). As early as more than a decade ago, Degenhardt et al. reported that autosis may suppress tumors by attenuating metabolic stress in apoptosis-deficient cells (158). In addition, autosis can act together with ferroptosis to exert anticancer effects in tumor cells. The AMPK signaling pathway activates BECN1, which promotes ferroptosis in tumor cells by directly blocking System Xc- and binding to SLC7A11 to improve anticancer therapy (159). The interaction with two pathways of GPX4 and mTOR can regulate autophagy-dependent ferroptosis in pancreatic cancer cells (160). Interestingly, autosis, in addition to its anticancer role, also plays a carcinogenic role in the early stage of tumors. Studies using a Drosophila melanogaster malignant tumour model have demonstrated that TNF and IL-6 mediated autophagy can modulate the tumor microenvironment and participate in tumor growth (161).

### 8.3 Autosis and lncRNAs in tumors

LncRNAs have been reported to affect autosis in tumor cells by regulating the ULK1 complex and mTOR signaling pathway. Zhao et al. found that lncRNA H19 can impact on glioma cell proliferation, migration, and autophagy by regulating the mTOR signaling pathway. Overexpression of lncRNA H19 inhibits mTOR phosphorylation and promotes ULK1 phosphorylation, thereby inhibiting the development of autosis and promoting tumor cell proliferation (162). LncRNA H19 has also been reported in pituitary tumor (163). LncRNA H19 suppresses mTORC1 function and blocks mTORC1-mediated phosphorylation of 4E-BP1 to inhibit pituitary tumor cell proliferation in vitro and in vivo (163). LncRNA CASC9 can promote tumor proliferation by inhibiting autosis and autophagy-mediated apoptosis through AKT/mTOR pathway in oral squamous cell carcinoma (164). There are two pathways by which lncRNA HAGLROS regulates the mTOR signaling pathway in gastric cancer. One is to increase the expression of mTOR mRNA and mTOR by sponging miR-100-5p; the other is that lncRNA HAGLROS interacts with mTORC1 components to activate the mTORC1 signaling pathway and inhibit autosis in gastric cancer cells (165). LncRNA EPIC1 can activate AKT-mTORC1 signaling pathway through regulating the expression of transcription factor Myc, which leads to rapamycin resistance and reduced autosis in tumor cells in breast and ovarian cancers (166). In addition to ULK1, two other proteins in the ULK1 complex have not been covered to be regulated by lncRNAs.

There are quite a few reports on the regulation of autophagy by lncRNAs, but there are few reports on the involvement of lncRNAs in the regulation of autophagy-dependent cell death, and there is still much room to be explored on the relationship between lncRNAs and autophagy-dependent cell death.

## 9 Cuproptosis

### 9.1 Mechanisms of cuproptosis

Cuproptosis belongs to RCDs discovered in 2022. Tsvetkov and colleagues showed that intracellular copper accumulation leads to aggregation of lipoylated proteins and destabilization of Fe-S cluster proteins in mitochondria, which in turn induces a unique type of cell death named cuproptosis (167). Changes in cell morphology have not been reported when cuproptosis occurs.

Elesclomol and copper ion complex (ES-Cu) can induce cuproptosis. ES in ES-Cu complexes can promote Cu^2+^ into cells. Ferredoxin 1 (FDX1) is a direct target of ES, and Cu^2+^ are reduced to Cu^+^ by FDX1 (168). FDX1 is a key component in the Fe-S cluster assembly pathway and also involved in lipoylation of the tricarboxylic acid (TCA) cycle proteins (169). TCA cycle proteins that can be lipoylated include line dihydrolipamide branched-chain transacylase E2, glycine cleavage system protein H, dihydrolipamide S-succinyltransferase, and dihydrolipamide S-acetyltransferase (DLAT), which are important components of the pyruvate dehydrogenase complex (170, 171). Cu^+^ reduced by FDX1 can combine directly with lipoylated DLAT and promote disulfide-bond-dependent DLAT oligomers formation. Meanwhile, FDX1-dependent Fe-S cluster proteins undergo degradation, causing cuproptosis in cells (167). (**Figure 4**)

### 9.2 Cuproptosis in tumor cells

As a key molecule of cuproptosis, serum copper has been reported to be more abundant in lung cancer, hepatocellular carcinoma, colorectal cancer, breast cancer, cervical cancer, oral cancer and other tumors compared with normal tissues, and copper is considered to be involved in tumor growth, metastasis, and drug resistance (172–178). A mitochondria-targeted, copper-depleting nanoparticle chelates copper in mitochondria, reduces oxygen consumption and oxidative phosphorylation, converts metabolism to glycolysis to reduce ATP production in triple-negative breast cancer cells, and ultimately inhibits tumor growth and improves survival rate (179). In addition, copper depletion activates AMP-activated protein kinase to inhibit mTORC1 pathway, and reduce oxidative phosphorylation which in turn weakens the ability of tumor invasion (180). The expression level of cuproptosis-related gene SLC31A1, i.e., copper transporter 1 (CTR1), is negatively correlated with overall survival. The study revealed that CTR1 is abnormally elevated in breast cancer and copper activates the PDK1/AKT pathway in a CTR1-dependent manner and promotes tumorigenesis (181). Apart from copper and SLC31A1, other cuproptosis-related genes, such as FDX1, ATP7B, and lipoyltransferase-1, are also involved in tumor development (182, 183). FDX1 which expression is significantly lower than normal tissues in a variety of cancer types, is positively correlated with immune cell infiltration and tumor mutation load, and has a clear correlation with tumor survival and prognosis. Therefore, FDX1 is expected to be a tumor biomarker and potential therapeutic target (169).

### 9.3 Cuproptosis and lncRNAs in tumors

After cuproptosis was reported, studies about lncRANs and cuproptosis-related genes have gradually increased, and have been reported in a variety of tumors. Mo et al. analyzed that AC008764.2, AL022323.1, lncRNA ELN-AS1 and LINC00578 are protective lncRNAs that promote cuproptosis in lung adenocarcinoma cells, while AL031667.3, AL606489.1 and lncRNA MIR31HG are considered as dangerous lncRNAs (184). Among them, lncRNA MIR31HG can inhibit cuproptosis and promote the proliferation, migration, and invasion of lung adenocarcinoma cells by down-regulating miR-193a-3p and increasing downstream TNFRSF21 expression (184). In osteosarcoma, AL645608.6, AL591767.1, lncRNA UNC5B-AS1, lncRNA CARD8-AS1, AC098487.1, AC005041, lncRNA TIPARP-AS1, lncRNA RUSC1-AS1, and LINC02315 play a role in regulating cuproptosis (185, 186). The studies on the regulatory relationship of lncRNAs on cuproptosis in breast cancer have also been reported. Jiang et al. found that lncRNAs such as lncRNAs GORAB-AS1, AC079922.2, AL589765.4, AC005696.4, lncRNA Cytor, lncRNA ZNF197-AS1, AC002398.1, AL451085.3, lncRNA YTHDF3-AS1, AC008771.1, and LINC02446 are associated with cuproptosis and can affect the prognosis of breast cancer and the sensitivity of immunotherapy (187). LncRNA XIST can sponge miR-92b-3p and regulate the cuproptosis-related gene MTF1 to influence the progression of breast cancer (188). In cervical cancer, AL441992.1, LINC01305, AL354833.2, lncRNA CNNM3-DT and lncRNA SCAT2 can promote cuproptosis to protect the body from tumor cells attack and improve tumor prognosis; while AL354733.3 and AC009902.2 can inhibit cuproptosis to facilitate tumor growth (189). In addition, there are many relevant reports on lncRNAs regulating cuproptosis in head and neck squamous cell carcinoma, gastric cancer, liver cancer, and colorectal cancer (190–193).

However, the regulatory relationship between lncRNAs and cuproptosis is only analyzed in the database, but there are fewer experimental studies about the two relationships. Therefore, it remains many doubts whether lncRNAs can regulate cuproptosis in tumor cells, which needs further exploration.

## 10 Discussion

LncRNAs have been the research focus in recent years, especially in the regulation of tumor biological function. LncRNAs can initiate RCDs mediated by different pathways which act on tumor cells for the purpose of promoting or inhibiting tumor proliferation, migration, invasion, prognosis, and drug resistance. The occurrence of a particular RCD is often regulated by many different lncRNAs in the same tumor. For example, lncRNA PDPK2p/PDK1/AKT/caspase-3 signaling pathway can inhibit apoptosis, while the lncRNA MAGI2-AS3/miR-374b-5p/Smg1 axis can promote apoptosis in liver cancer cells (38, 41). In cervical cancer, lncRNA FAM13A-AS1/miRNA-205-3p/DDI2 axis and lncRNA PTENP1/miR-106b/PTEN axis can jointly promote apoptosis and inhibit the progression of cervical cancer, while lncRNA HOXD-AS1 plays an anti-apoptosis role to promote the progression of cervical cancer (194–196). In addition to apoptosis, different lncRNAs have also been reported to regulate the same tumor in other RCDs such as necroptosis and ferroptosis. Homologous lncRNAs can play different roles in different tumors and initiate different RCDs. LncRNA H19 has been reported to regulate apoptosis, ferroptosis, and others (197). LncRNA NEAT1 regulates pyroptosis in glioma cells and colorectal cancer cells by targeting miR-296-5p or miR-448; while its high expression promotes ferroptosis in non-small cell lung cancer (76, 77, 135). Furthermore, lncRNA H19 and lncRNA NEAT1 act upon each other and respectively regulate apoptosis by targeting miR-675 and miR-204 in breast cancer (198). Although lncRNAs have been demonstrated successively as potential targets for tumor diagnosis and treatment, there is no mature means to use lncRNAs for clinical diagnosis and treatment, which is still a great gap waiting to be explored.

RCDs can occur on almost all cells and play indispensable roles in the normal growth and development of the human body. The signaling pathways of RCDs can interact with each other. As a very vital connection in the process of apoptosis, caspase family also play a part in necroptosis and pyroptosis; ROS is not only working in ferroptosis, but also participates in NETosis. In addition, some forms of cell death can also act depending on other forms of cell death. Apoptosis, which is dependent on caspase, can stimulate GSDM cleavage through caspase activation and alter GSDM expression in triggering pyroptosis (199). LINC00618 exerts apoptosis by upregulating levels of Bax and caspase-3, while LINC00618 induces ferroptosis by increasing ROS and decreasing SLC7A11. Findings used caspase inhibitors Z-VAD-FMK and erastin demonstrated that LINC00618-induced ferroptosis was dependent on apoptosis (147). However, the specific mechanism by which lncRNAs promote or inhibit different RCDs in tumor cells through targeting common key molecules that regulate multiple pathways remains unreported.

Finally, due to the limited available medical technology, people still do not conquer cancers. It is difficult to diagnose malignant tumors in time and provide effective and specific treatment for a long time, resulting in the prognosis of most malignant tumors is still not optimistic. Therefore, we need a target that is specific and sensitive, and lncRNAs may become this target. LncRNAs can improve tumor prognosis by triggering different RCDs, which provides us with new ideas for diagnosis and treatment.

## Author contributions

Conceptualization, YL and YW; manuscript preparation, YW; manuscript revision, YL, YW, XW, YX and XY. All authors have read and agreed to the published version of the manuscript.
